# Characterization of the Novel Phage vB_VpaP_FE11 and Its Potential Role in Controlling *Vibrio parahaemolyticus* Biofilms

**DOI:** 10.3390/v14020264

**Published:** 2022-01-27

**Authors:** Meiyan Yang, Hanfang Chen, Qiaolan Huang, Zhuanbei Xie, Zekun Liu, Jumei Zhang, Yu Ding, Moutong Chen, Liang Xue, Qingping Wu, Juan Wang

**Affiliations:** 1 Guangdong Provincial Key Laboratory of Microbial Safety and Health, State Key Laboratory of Applied Microbiology Southern China, Institute of Microbiology, Guangdong Academy of Sciences, Guangzhou 510070, China; ymy@scau.edu.cn (M.Y.); chenhanfangh@163.com (H.C.); xiezhuanbei@163.com (Z.X.); liuzekun98@gmail.com (Z.L.); zhangjm926@126.com (J.Z.); cmtoon@hotmail.com (M.C.); xueliang@gdim.cn (L.X.); 2 College of Food Science, South China Agricultural University, 483 Wushan Road, Guangzhou 510642, China; 3 College of Agriculture, South China Agricultural University, 483 Wushan Road, Guangzhou 510642, China; symxmyz3133@163.com; 4 Department of Food Science & Technology, Institute of Food Safety and Nutrition, Jinan University, Guangzhou 510642, China; dingyu@jnu.edu.cn

**Keywords:** *Vibrio parahaemolyticus*, aquaculture, biofilm, phage, biological control

## Abstract

*Vibrio parahaemolyticus* causes aquatic vibriosis. Its biofilm protects it from antibiotics; therefore, a new different method is needed to control *V. parahaemolyticus* for food safety. Phage therapy represents an alternative strategy to control biofilms. In this study, the lytic *Vibrio* phage vB_VpaP_FE11 (FE11) was isolated from the sewers of Guangzhou Huangsha Aquatic Market. Electron microscopy analysis revealed that FE11 has a typical podovirus morphology. Its optimal stability temperature and pH range were found to be 20–50 °C and 5–10 °C, respectively. It was completely inactivated following ultraviolet irradiation for 20 min. Its latent period is 10 min and burst size is 37 plaque forming units/cell. Its double-stranded DNA genome is 43,397 bp long, with a G + C content of 49.24% and 50 predicted protein-coding genes. As a lytic phage, FE11 not only prevented the formation of biofilms but also could destroy the formed biofilms effectively. Overall, phage vB_VpaP_FE11 is a potential biological control agent against *V. parahaemolyticus* and the biofilm it produces.

## 1. Introduction

*Vibrio parahaemolyticus*, a halotolerant Gram-negative bacterium, is an important food-borne pathogen commonly present in aquatic products [1,2]. With the consumption of uncooked seafood becoming a growing trend, the risk of infection with *V. parahaemolyticus* has increased. The clinical symptoms of *V. parahaemolyticus* infections include abdominal pain, diarrhea, nausea, vomiting, and fever [3,4]. Furthermore, *V*. *parahaemolyticus* can cause substantial economic losses in aquaculture industry [5].

Biofilms are important for bacterial growth and survival; *V. parahaemolyticus* uses them to survive in natural or food-processing environments [6]. Bacterial cells in biofilms are more resistant to antibiotics than planktonic bacteria [7]; hence, *V. parahaemolyticus* biofilms pose a major threat to food safety.

Bacterial resistance to antibiotics has become increasingly common with the overuse of the latter [8,9]; therefore, it is necessary to develop new effective antimicrobial agents. Bacteriophages, the most abundant biological entities on Earth, reduce the global bacterial population by half every 48 h via phage predation [10]. In addition, phages are natural entities and highly specific; hence, using them as antimicrobial agent is cost effective. Studies on phage therapy have demonstrated its potential as an alternative to antibiotic therapy; for example, phages can be used to control *Vibrio* infection in the culture of aquatic organisms such as sea cucumbers [11,12] and Atlantic salmon [13]. Phage therapy has also been used to control the formation of *Vibrio* biofilms [14,15].

In this study, a *V. parahaemolyticus* phage was isolated from sewage. The phage was preliminarily classified by morphology using transmission electron microscopy (TEM). BLASTn and ANIm were used to compare the nucleotide sequence and combined with the results of TEM to further classify the phage. Phylogenetic trees were used to determine the phylogenetic relationships of phage. Finally, the phage was classified at the genus level using vConTACT. There are few studies on the control of *V. parahaemolyticus* biofilms by phages. We investigated the control effects of phage vB_VpaP_FE11 (FE11) on *V. parahaemolyticus* biofilms in vitro. These results will broaden our knowledge about *V. parahaemolyticus* phage and potentially provide a theoretical basis with which to treat *V. parahaemolyticus* biofilms.

## 2. Materials and Methods

### 2.1. Bacterial Strains and Growth Conditions

All *V*. *parahaemolyticus* strains used in this study were provided by the Institute of Microbiology, Guangdong Academy of Sciences and were stored at −80 °C in 30% (*v*/*v*) glycerol (Table 1). *V. parahaemolyticus* strain O5–15 (VP O5–15, antigen O5 and strain no. 15) was isolated from infected shrimp and incubated at 37 °C in tryptic soy broth (TSB) while being shaken at 200 rpm overnight.

### 2.2. Phage Isolation and Purification

Phages were isolated from the sewage taken from Huangsha Aquatic Market in Guangzhou. The sewage sample was centrifuged at 5000× *g* for 10 min, following which the supernatant was suction-filtered using the 0.45 μm (47 mm diameter) mixed cellulose ester GSWP filter (HuanKai Microbial, Guangzhou, China). Thereafter, MgSO_4_ was added to a final concentration of 50 mM for phage particles gathering, the mixture was allowed to stand for 10 min before being suction-filtered again using a 0.22-μm filter [16]. The filter membranes were cut and eluted with a broth containing 3% (*w*/*v*) Bacto beef extract, 3% (*v*/*v*) Tween 80 and 50 mM NaCl. Subsequently, 2 mL of eluent and 100 µL of early log-phase VP O5–15 cultures were mixed with 2 mL of double-strength TSB supplemented with 4 mM CaCl_2_, and incubated overnight at 37 °C while being shaken (200 rpm). The culture was then filtered through the 0.45 μm syringe filter. The process was repeated three times. Phage presence was verified using a double-layered plate. A suitable dilution of phage (approximately 1 × 10^3^ plaque forming units (pfu)/mL, 100 μL) was mixed with 100 μL of VP O5–15, added to 5 mL TSB with 0.4% agar (2 mM CaCl_2_), poured onto a 1.5% TSB agar plate, and incubated at 37 °C for 5 h. Finally, a single isolated plaque was selected and purified at least six times. The purified phage was stored at 4 °C for later use.

### 2.3. Transmission Electron Microscopy (TEM) Analysis

The method of Ajuebor et al. [17] was followed with minor modifications. Before TEM analysis, the phage was concentrated using gradient centrifugation. Cesium chloride solutions (2.5 mL of 1.3 g/mL, 1.5 g/mL, and 1.7 g/mL) were layered sequentially to a 10-mL Beckman centrifuge tube to which 2.5 mL of the concentrated phage solution was added. Thereafter, the solution was centrifuged using a SW 41 Ti rotor (Optima XPN–100 ultracentrifuge, Beckman Coulter, Brea, CA, USA) at 200,600× *g* for 3 h at 4 °C. After centrifugation, the phages formed a bluish-white band at the interface between 1.3 and 1.5 g/mL CsCl [18]. The phage particles were collected by suctioning with a syringe placed just underneath the target band. The collected phages were negatively stained with 2% (*w*/*v*) phosphotungstic acid on a carbon-coated grid and examined using a transmission electron microscope (Hitachi H–7650, Tokyo, Japan).

### 2.4. Phage Host Range

*V*. *parahaemolyticus* has been classified to 13 types based on its LPS O antigens [19]. Phage host range was determined using 133 *V. parahaemolyticus* strains that represent the different O types. First, 100 μL of early log-phase bacterial culture was mixed with 5 mL of warm soft agar (TSB containing 0.4% agar, 2 mM CaCl_2_) and poured onto 1.5% TSA plates, following which 2 µL drops of 10-fold diluted phage stock were pipetted on each plate. Thereafter, the plates were incubated at 37 °C and observed after 5 h. Phage hosts were confirmed by the appearance of distinct plaques on the plate.

### 2.5. Isolation, Genome Sequencing, and Assembly of Phage DNA

Phage DNA was extracted according to Zhao et al. [20,21] with some modifications. Briefly, the phage was precipitated overnight with 15% (*w*/*v*) PEG 8000 and 0.5 M NaCl at 4 °C. Subsequently, the phage was centrifuged at 12,000× *g* for 20 min and then resuspended in SM buffer. The concentrated phage particles were treated with DNase I (final concentration 0.1 units/μL) and RNase A (final concentration 3 μg/mL) to remove bacterial genomic contamination. Thereafter, the sample was treated with SDS, EDTA, and proteinase K. Finally, the phage DNA was extracted using phenol-chloroform-isoamyl alcohol (25:24:1). The DNA was sequenced using the Ion Torrent S5 platform (Thermo Fisher Scientific, Waltham, MA, USA) and high-quality reads were subsequently assembled using SPAdes v. 3.12.0.

### 2.6. Genome Analysis and Phylogenetic Analysis

PhageTerm was used to identify phage termini and reassemble the whole genome sequence of FE11 [22]. The genome data was analyzed using the National Center for Biotechnology Information (NCBI) database (https://blast.ncbi.nlm.nih.gov/Blast.cgi accessed on 18 November 2021). The genome sequences of 24 *Podoviridae* members of *Vibrio parahaemolyticus* were downloaded from NCBI. Similarities between genomic sequences were determined using the average nucleotide identity MUMmer (ANIm) [23]. The heatmap was drawn using TBtools. Putative transfer RNA (tRNA)-encoding genes were predicted using tRNAscan-SE. Predicted virulence factors and antibiotic genes were examined using searches against the Virulence Factor Database and the Antibiotic Resistance Gene Database, respectively. The complete genome was automatically annotated by Prokka. Further analysis of the predicted gene products was conducted using BLASTP [24], InterProScan [25] and HHpred [26]. Groups of similar phage genomes were visualized using Easyfig. Phylogenetic analysis based on RNA polymerase was conducted using the neighbor-joining method in MEGA X with 1000 bootstrap replicates [27]. Finally, taxonomic assignment of phage genomes was performed using vConTACT [28] and visualized using the software Cytoscape.

### 2.7. One-Step Growth Experiment

The overnight culture of VP O5–15 was diluted 1:50 to a fresh medium (TSB) and cultured at 37 °C, 200 rpm for 1 to 2 h, during which the bacterial cells in 0.1 mL of the medium were counted every 10 min using a hemocytometer. The optical density at 600 nm (OD_600_) of the suspension was also measured. A standard curve was generated and the corresponding viable count was estimated using OD_600_. A one-step growth curve was generated following a previously described method [29]. Briefly, VP O5–15 was cultured at 37 °C to the early-exponential growth-phase. One milliliter of the bacterial suspension (1 × 10^8^ colony forming units (cfu)/mL) was centrifuged at 8000× *g* for 5 min, and the pellet was resuspended in 1 mL of SM buffer (2 mM CaCl_2_). The phage was added at multiplicity of infection (MOI) = 0.1, the mixture was swirled gently and incubated at 37 °C for 10 min. The mixture was centrifuged at 12,000× *g* for 2 min to remove free phage, and the pellet was suspended in 1 mL of TSB. Thereafter, 0.1 mL of this solution was mixed with 9.9 mL TSB medium (2 mM CaCl_2_); this step corresponded to time zero (T_0_). Next, the mixed medium was cultured for 40 min at 37 °C, 200 rpm. The sample was obtained every 5 min interval to determine the titer of FE11. The experiment was repeated three times. The burst size of the phage was calculated as follows: burst size = the final count of phage / (initial count of phage-titer at T_0_).

### 2.8. Effects of Temperature, pH, and Ultraviolet (UV) Irradiation on Phage Activity

To investigate the stability of FE11, the effects of temperature, pH, and UV on phage activity were evaluated as described in another study [30,31]. First, the phage suspensions (1.0 × 10^8^ pfu/mL were incubated in a water bath at 20, 37, 40, 50, 60, and 70 °C; phage tittering was conducted after 1 h of incubation. HCl and NaOH were used to adjust the different pH (3, 4, 5, 6, 7, 8, 9, 10, 11, and 12) of TSB. Phages were equally added to this TSB and placed for 1 h at 37 °C. To determine the impact of ultraviolet (UV) irradiation, the phage suspension was exposed to UV light (254 nm and 25 W) and phage titering was carried out every 5 min for 30 min. Each experiment was repeated three times.

### 2.9. Effect of Phages on the Biofilm Formed

Previously reported methods [32,33] were used, with some modifications, to determine the effect of phages on the biofilm formed. *V*. *parahaemolyticus* (2.0 × 10^6^ cfu/mL) was distributed in 200 µL aliquots to the wells of a 96-well microtiter plate, and cultured at 28 °C for 12 h. Thereafter, the medium was removed and each well was washed three times with 200 μL of sterile phosphate-buffered saline (PBS). Next, 200 μL of FE11 (1 × 10^10^, 1 × 10^9^, 1 × 10^8^, 1 × 10^7^, 1 × 10^6^, 1 × 10^5^, 1 × 10^4^, and 1 × 10^3^ pfu/mL) and TSB was added; for the control group, 200 μL of TSB was added. The samples were cultured at 28 °C for 1, 2, 3, 4, and 5 h. At specific times, one plate was taken out, the medium was removed, and each well was washed three times with 200 μL of sterile PBS. Next, 200 μL of methanol was added to each well and incubated for 20 min before removal. After being air-dried, 200 μL of 0.1% crystal violet was added to each well and incubated for 15 min. Excess staining was removed by rinsing with water; thereafter, the plates were dried at 37 °C. Subsequently, 33% *v*/*v* glacial acetic acid was added for elution and the OD_590_ was measured using a microplate reader (BioTek Instruments Inc., Winooski, VT, USA).

### 2.10. Effect of Phages on the Formation of Biofilm

This experiment used the method described in Section 2.9 with some modifications. Phage FE11 (100 μL) was added to the VP O5–15 culture (1.0 × 10^8^ cfu/mL) at the MOIs of 100, 10, 1, 0.1, 0.01, 0.001, and 0.0001; SM buffer (100 μL) was added to the bacterial culture as a control. A total of 200 μL of the culture was added to 96-well plates. Three identical plates were prepared together and cultured at 28 °C for 6, 9, and 12 h, respectively. Subsequently, the OD_590_ was measured as specified in Section 2.9.

### 2.11. Scanning Electron Microscopy (SEM) Analysis

A previously described method [34] was used, with some modifications, for the SEM analysis in which 200 μL of phage FE11 (1 × 10^10^ pfu/mL) and 200 μL of VP O5–15 (1 × 10^8^ cfu/mL, MOI = 100) were added to a 48-well plate with 8 mm cell slides at the bottom. The control group had equal amounts of VP O5–15 and TSB. The plates were incubated at 37 °C, 12 h. Next, the cell slide was removed using a pair of tweezers and washed twice with 1×PBS. Each slide was then immobilized with 3% pentanediol at 4 °C for 5 h, after which they were dehydrated in a gradient ethanol series (30%, 50%, 70%, 90%, and 100%), and freeze-dried. Finally, the amount and morphology of biofilm were observed using a scanning electron microscope (Hitachi S-3000 N, Tokyo, Japan).

### 2.12. Statistical Analysis

The data were expressed as mean ± Standard deviation (SD) and the differences were analyzed with two-way ANOVA using GraphPad Prism 7.0. Significance was considered at *p* < 0.05.

### 2.13. Accession Number

The whole genome sequence of phage vB_VpaP_FE11 has been deposited at GenBank under the accession number MT178448.

## 3. Results

### 3.1. Isolation and Morphological Characterization of Phage FE11

**Figure 1:**
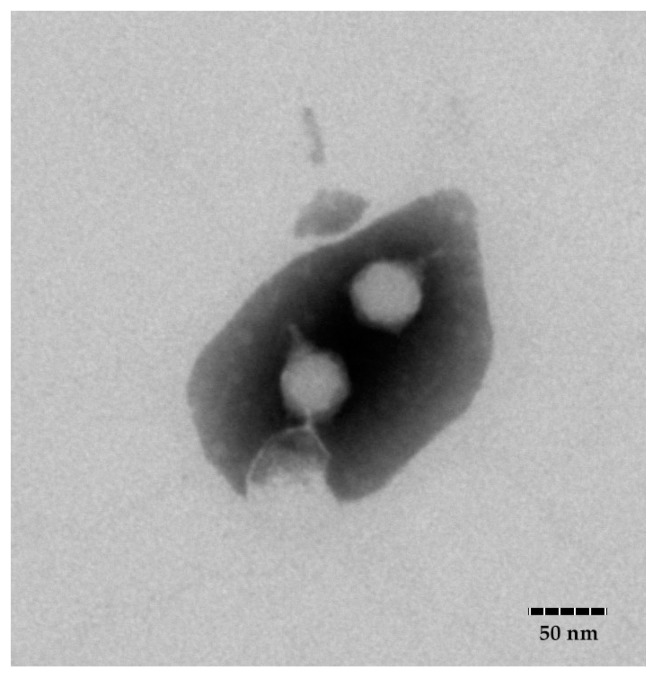
TEM images of vB_VpaP_FE11 (FE11), scale bar = 50 nm.

TEM analysis (Figure 1) showed that FE11 consist of an icosahedral head (diameter 47 ± 2 nm) and a short, non-contractile tail (length 18 ± 2 nm). This indicated that the phage belonged to the family *Podoviridae*. The phage was named as *Vibrio* phage vB_VpaP_FE11 as per international nomenclature [35].

### 3.2. Host Range of Phage FE11

The 133 strains of *V. parahaemolyticus* listed in Table 1—including the strain VP O5–15, which was used to isolate phages—were used to determine the antimicrobial spectrum of phage FE11. Host range was determined using spot assays. We found that FE11 could infect 35 of the 133 strains of *V. parahaemolyticus*. Phage FE11 host range was distributed among most of the 11 O-serotypes of *V. parahaemolyticus* tested in this study, suggesting that the O-antigen was not the specific receptor of the phage. Interestingly, among the serotype O5 strains it could only infect the original host strain VP O5–15.

### 3.3. The Genome Analysis of FE11

**Figure 2:**
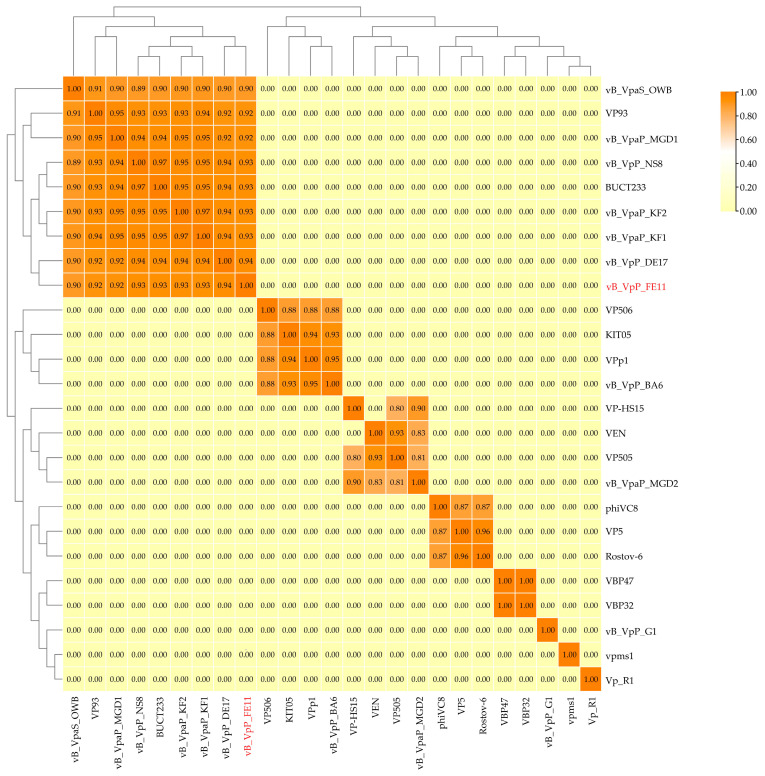
Average nucleotide identity heatmap. The percentage identity values range from 0 (0%, yellow) to 1 (100%, orange).

**Figure 3:**
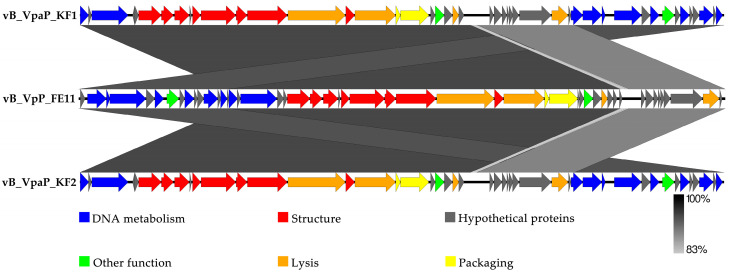
Comparison of the genomes of FE11, and vB_VpaP_KF1, and vB_VpaP_KF2 using Easyfig. Different colored arrows represent 50 predicted open reading frames with different functions.

The PhageTerm results indicated that FE11 has redundant ends and headful (pac) phage packaging (Figure S1). The genome of phage FE11 is 43,397 bp long and consists of linear double-stranded DNA with a GC content of 49.24%. It had the highest sequence similarity with the genomes of phage vB_VpaP_KF1 (identity × coverage = 93%) and vB_VpaP_KF2 (identity × coverage = 92.9%) by BLASTn. The ANI heatmap showed that FE11 is distinct from the other phages (Figure 2). The ANIm percentage identity of FE11 with vB_VpaP_KF1 (KF1) and vB_VpaP_KF2 (KF2) were both 93%. Among the 50 predicted genes, 25 showed similarities to genes encoding proteins of known function (Figure 3, Table S1). The remaining 25 predicted genes encoded hypothetical proteins. The FE11 proteins with predicted functions could be categorized into five functional groups: DNA metabolism, structure, lysis, DNA packaging and other function group, which respectively contained 9, 8, 4, 2 and 2 proteins. The predicted RNA polymerase was included in the DNA metabolism group. No tRNA genes were found using the tRNAScan−SE analysis, suggesting that FE11 depends on host translation. Furthermore, there were no virulence and antibiotic resistance genes in the genome of FE11, indicating that it could be safely used to control *V. parahaemolyticus*.

**Figure 4:**
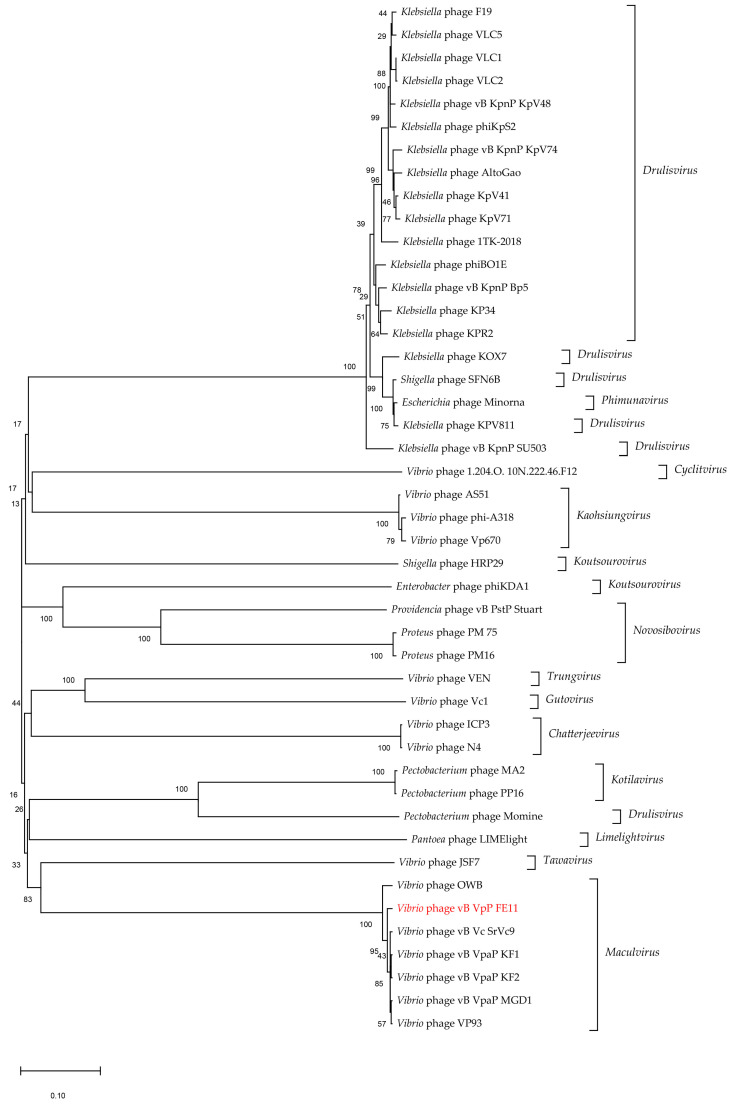
Phylogenetic tree of *Vibrio* phages in the subfamily *Autographiviridae*, conducted using the neighbor-joining method in MEGAX by RNA polymerases.

**Figure 5:**
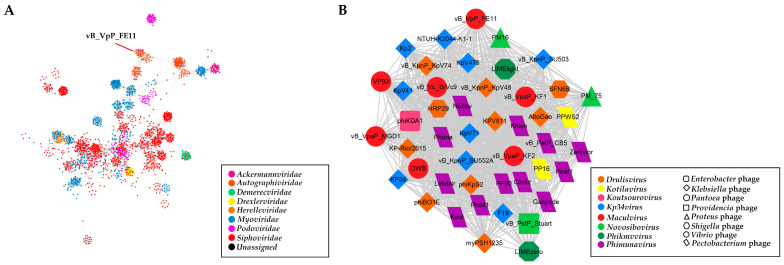
Protein-sharing network of FE11 benchmarked against ICTV-accepted viral taxonomy. (**A**) The network consists of 3445 phages (nodes) and 80,374 relationships (edges). Each node represents a viral genome, and each distinct color represents the viral family. (**B**) The protein cluster where FE11 was located. Different shapes represent phages with different hosts and each distinct color represents the viral genus.

The phage particle morphology and the analysis of the functions of the predicted gene products allowed to conclude that FE11 is a typical member of the *Podoviridae*. The fact that it has its own RNA polymerase places it, according to the ICTV classification, to the subfamily *Autographivirinae*. Therefore, the genome sequence of 44 *Autographivirinae* phages were downloaded from the ICTV database for phylogenetic analysis. The results (Figure 4) showed that FE11, vB_Vc_SrVc9, KF1, and KF2, were clustered in the same branch; this was consistent with the results of the whole genome similarity analysis. Next, a protein-sharing network was analyzed to determine the exact taxonomic status of FE11. As shown in Figure 5, nodes represented the viral genomes and edges between the nodes represented the genetic similarities between them. A total of 3445 phages were selected from the vConTACT database for analysis. In Figure 5B, FE11 was marked with a red circle, indicating that it belongs to the genus *Maculvirus*.

### 3.4. One-Step Growth Curve

**Figure 6:**
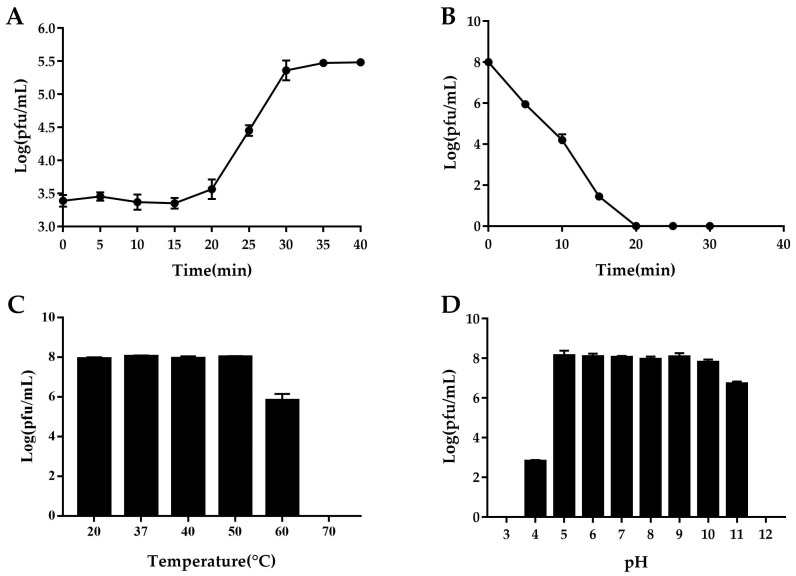
General characterizations of phage FE11. (**A**) One-step growth curve. (**B**) Effects of ultraviolet irradiation on phage activity. (**C**) Temperature tolerance. (**D**) pH tolerance.

A one-step growth curve was used to elucidate the life cycle of the phage. The results showed that the latent period of FE11 (Figure 6A) was 15 min. The lysis period was 15 min, when the number of phages increased markedly, then plateaued. The burst size was 37 pfu/cell.

### 3.5. Determination of Phage Stability

Under UV light, the phage survival rate decreased sharply at approximately 2 log values every 5 min. The phage was completely inactivated after 20 min of UV irradiation, indicating that FE11 was sensitive to UV light (Figure 6B).

The heat stability test showed that the phage titer remained almost unchanged between 20 to 50 °C, decreased markedly at 60 °C, and the phage was completely inactivated when the temperature reached 70 °C (Figure 6C).

The phage titer remained almost constant at pH values ranging from 5 to 10. Under acidic conditions, the titer decreased considerably at pH values from 5 to 4, and the phage was inactivated at pH 3. Under alkaline conditions, the phage titer decreased by approximately 1.1 logs as the pH increased from 10 to 11, and the phage was inactivated completely at pH 12 (Figure 6D).

### 3.6. Effect of Phage on Formed Biofilm

**Figure 7:**
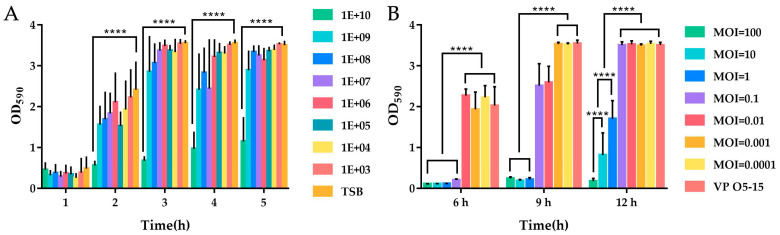
Effects of phage on biofilm. (**A**) Effects of FE11 of different titers on the preformed biofilm. Different treatments are indicated by different bar colors. ****, *p* < 0.0001. (**B**) Effects of phage co-incubation on biofilm formation. Shown are the results on the effects of FE11 at different MOIs on the biofilm formation after 6, 9, and 12 h of co-incubation. Control: *Vibrio parahaemolyticus* strain O5–15 (VP O5–15) without any phages. ****, *p* < 0.0001.

As shown in Figure 7A, most treatments were almost ineffective for formed biofilm; however, from 2 to 5 h, when the concentration of phages added was 1 × 10^10^ pfu/mL, the effect was significant (*p* < 0.01). This suggests that there must be a sufficient concentration of phages to destroy the formed biofilm.

### 3.7. Effect of Phage on Biofilm Formation

**Figure 8:**
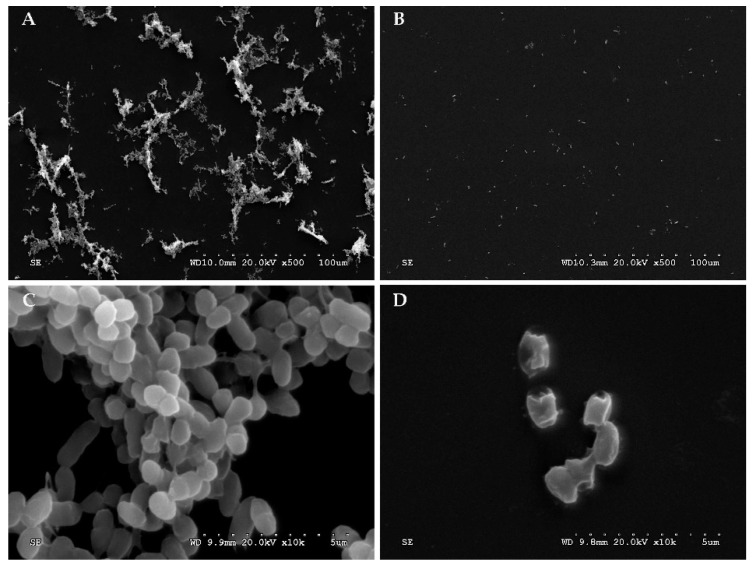
Scanning electron microscope imagery of *V. parahaemolyticus* O5–15 treated with phage FE11: (**A**,**C**) *V. parahaemolyticus* O5–15, and (**B**,**D**) *V. parahaemolyticus* O5–15 + FE11.

As shown in Figure 7B, the phage treatment could effectively control the formation of *V. parahaemolyticus* biofilm at a very low concentration at 6 h with MOI of 100, 10, 1, and 0.1 compared with the control and other MOIs (*p* < 0.01). With an MOI of 0.1, there was a significant increase of OD value from 6 h to 9 h, which meant the biofilm increased greatly and the effect of phage treatment decreased. When co-cultured for 12 h, treatments with MOI of 100, 10, and 1 remained effective, with MOI of 100 exhibiting the most significant effect (*p* < 0.01). The higher the MOI, the better the control and prevention effects of biofilm. The results of SEM analysis further confirmed that phages could effectively control the formation of *V. parahaemolyticus* biofilm. As shown in Figure 8A, VP O5–15 could easily form biofilms. After treatment with FE11, the amount of biofilm formed decreased markedly (Figure 8B). As shown in Figure 8C, the untreated VP O5–15 cells were complete and smooth, whereas the cells ruptured in the presence of FE11 (Figure 8D).

## 4. Discussion 

*V. parahaemolyticus* infects fish, shellfish, and shrimp and is one of the main causes of seafood-borne illnesses [36,37]. *V. parahaemolyticus* can form biofilms in environments where food is processed [38], which is a major food safety risk in the seafood and aquaculture industries. An increasing number of antibiotic-resistant strains of *V. parahaemolyticus* have been isolated [39,40]; therefore, an effective antibacterial agent is needed to counter this. Lytic phages have the potential for use in an antibacterial strategy [41,42,43].

This study identified a *Vibrio* phage capable of infecting 26% (35/133) of tested *V. parahaemolyticus* strains. Based on morphological observation and genome sequencing, a BLASTn search combined with ANIm analyses revealed that FE11 belongs to the family *Podoviridae*.

In the genome of FE11, Prokka annotation, BLASTp analysis, InterProScan, and HHpred analysis showed that the predicted gene products of FE11 are similar to those of phages KF1 and KF2, with a few differences; FE11 contains four additional predicted genes (*gene 01*, *34*, *40* and *41*) encoding hypothetical proteins. Annotation results showed that there were no genes related to lysogen formation, such as those encoding recombinases or integrases in the genome of FE11 further supporting the lytic nature of FE11.

The organization of FE11 genome showed modularity, such that genes with related function were clustered together. The upstream DNA region consisted of genes related to DNA metabolism, whereas the downstream region consisted of those related to packaging and lysis. Proteins related to DNA metabolism are involved in DNA replication and transcription. Predicted *gene 18* product is RNA polymerase, allowing the classification of FE11 to *Autographiviridae*. FE11 was further identified as *Maculvirus* based on phylogenetic analysis of RNA polymerase and protein family analysis by vConTACT.

The structural module of phage genome are always associated with host recognition, especially tail-related proteins. In the structure module of FE11 genome, three tail-related proteins were predicted, including tail tubular protein A/B (TTPA/TTPB, Gp25/26) and tail fiber protein (Gp30). The TTPA and TTPB of phage OWB have been demonstrated to serve as ligands that recognize the conserved *Vibrio* receptor Vp0980 to mediate phage adsorption [44]. The tail fibre of phage OWB binds LPS and mediates phage infection. So phage OWB requires both tail fiber and tail tubular proteins for host recongnition. The amino acid sequence of FE11 TTPA, TTPB and tail fiber have high similarity to that of phage OWB (93.01%, 94.62% and 96.06% identity, respectively). So we refer that the tail fiber and tail tubular proteins of phage FE11 mediate phage adsorption to host as well. But further studies are required to prove the function.

In phages, endolysin can lyse the peptidoglycan of the bacterial cell wall, thereby assisting in the release of new phages. Owing to its broad-spectrum lytic nature and the low possibility of bacterial resistance developing, endolysin has been used widely as part of antibacterial therapy [45,46,47]. As the outer membrane of Gram-negative bacteria hinders the permeation of exogenous endolysin, outer membrane permeabilizers (OMPs) are essential for combined use in in vitro treatment [48]. The peptidase (KF2_Lys) purified from *V. parahaemolyticus* phage KF2 reportedly showed a high lytic activity independent of OMP [49]. The predicted peptidase (Gp49) of FE11 is 93.19% identical with the KF2_Lys strongly indicateing that the two have similar functions. Further investigations to elucidate the lytic mechanism of FE11 endolysin are warranted.

In this study, an Ig-like domain family protein (Gp36) was identified in the FE11 genome; these proteins may help phages attach to cell surfaces or eliminate pathogenic bacteria invading mucosa by binding to the mucosal surface [50].

In terms of biological characteristics, FE11 was stable at a relatively broad temperature range between 20 and 50 °C, while phage vB_Vc_SrVc9 was only stable at 20−40 °C, which both phages were located in the same evolutionary branch [51]. For the UV test, FE11 was completely inactivated by UV irradiation for 20 min, but vB_Vc_SrVc9 was more sensitive and basically inactivated after 1 min. FE11 was also stable at relatively broad pH range, suggesting that it has potential for use in aquaculture environments.

Biofilms are aggregates of bacterial cells, and their heterogeneity leads to nutrient limitation and a decrease in metabolic activity and growth rate, thereby reducing the sensitivity to antibiotics [52]. There are many studies on the application of phages to prevent and control kinds of bacterial biofilms. For example, phages were able to reduce the biofilm formation of Shiga toxin-producing *Escherichia coli* by 43.46% [53]. Moreover, phages prevented the formation of methicillin-resistant *Staphylococcus pseudintermedius* biofilms at low doses, and even degrade the biofilms at high doses [54]. However, there were few studies on *V. parahaemolyticus* biofilms. The phage ϕVP−1 has been reported to control *V. parahaemolyticus* biofilms effectively [55]. Yin et al. showed that phages could prevent and control *V. parahaemolyticus* biofilms, but could not effectively destroy formed biofilms [56]. For phage relative productions, the endolysin of phage qdvp001 (Lysqdvp001−15 aa) could also reduce the biofilms of *V. parahaemolyticus* and inhibit the formation of the bacterial biofilms [57]. In our study, FE11 not only prevents the formation of *V. parahaemolyticus* biofilms, but also destroy the preformed biofilms at high phage concentrations.

In conclusion, the newly isolated *Vibrio* phage FE11 has the potential to become a biocontrol agent against *V. parahaemolyticus*.

## Figures and Tables

**Table 1:** Host range for FE11 against *Vibrio parahaemolyticus* (VP) strains.

VP Strains	Serotype	FE11	VP Strains	Serotype	FE11	VP Strains	Serotype	FE11	VP Strains	Serotype	FE11
O1–1	O1	−	O2–91	O2	+	O4–112	O4	−	O10–127	O10	−
O1–2	O1	+	O2–92	O2	−	O4–113	O4	+	O10–128	O10	−
O1–3	O1	+	O2–93	O2	−	O4–114	O4	+	O10–129	O10	−
O1–4	O1	−	O2–94	O2	−	O4–115	O4	−	O10–130	O10	−−
O1–36	O1	−	O2–95	O2	−	O5–15	O5	+	O10–131	O10	−
O1–37	O1	−	O2–96	O2	−	O5–16	O5	−	O10–132	O10	−
O1–38	O1	−	O2–97	O2	+	O5–17	O5	−	O11–29	O11	−
O1–62	O1	−	O2–98	O2	−	O5–49	O5	−	O11–30	O11	+
O1–63	O1	+	O2–99	O2	−	O5–50	O5	−	O11–31	O11	+
O1–64	O1	+	O2–100	O2	−	O5–116	O5	−	O11–56	O11	−
O1–65	O1	−	O2–101	O2	+	O5–117	O5	−	O11–57	O11	−
O1–66	O1	−	O2–102	O2	−	O5–118	O5	−	O11–58	O11	−
O1–67	O1	+	O2–103	O2	−	O6–18	O6	+	O11–133	O11	+
O1–68	O1	−	O3–8	O2	−	O6–19	O6	+	O11–134	O11	+
O1–69	O1	−	O3–9	O2	−	O6–20	O6	+	O11–135	O11	+
O1–70	O1	−	O3–10	O3	−	O6–120	O6	−	O11–136	O11	−
O1–71	O1	−	O3–11	O3	−	O8–21	O8	+	O11–137	O11	+
O1–72	O1	+	O3–42	O3	−	O8–22	O8	−	O11–138	O11	−
O1–73	O1	−	O3–43	O3	−	O8–51	O8	−	O12–32	O12	−
O2–5	O2	−	O3–44	O3	−	O8–52	O8	−	O12–33	O12	+
O2–6	O2	−	O3–104	O3	+	O8–53	O8	−	O12–34	O12	−
O2–7	O2	−	O3–105	O3	−	O8–121	O8	−	O12–35	O12	−
O2–39	O2	−	O3–106	O3	+	O8–122	O8	+	O12–60	O12	−
O2–40	O2	−	O3–107	O3	+	O8–123	O8	−	O12–61	O12	−
O2–41	O2	−	O4–12	O4	+	O8–124	O8	−	O12–140	O12	−
O2–82	O2	−	O4–14	O4	+	O8–125	O8	−	O12–141	O12	−
O2–83	O2	−	O4–45	O4	−	O8–126	O8	−	O12–143	O12	−
O2–84	O2	−	O4–46	O4	−	O9–24	O9	−	O12–144	O12	−
O2–85	O2	−	O4–47	O4	+	O10–25	O10	−	O12–145	O12	−
O2–86	O2	−	O4–48	O4	−	O10–26	O10	−	O12–146	O12	+
O2–87	O2	−	O4–108	O4	−	O10–27	O10	+	O12–147	O12	−
O2–88	O2	−	O4–109	O4	+	O10–28	O10	−			
O2–89	O2	−	O4–110	O4	−	O10–54	O10	+			
O2–90	O2	−	O4–111	O4	+	O10–55	O10	−			

## Data Availability

The findings of this study are available within this paper and its supplementary information file. The complete genome sequence of phage vB_VpaP_FE11 was submitted to the GenBank database under accession number MT178448.

## Supplementary Materials

The following are available online at https://www.mdpi.com/article/10.3390/v14020264/s1, Figure S1: The phage genome termini analysis of FE11, Table S1: Predicted functions of phage FE11 gene products.
