# Supplementary material for: Characterization of the Novel Phage vB_VpaP_FE11 and Its Potential Role in Controlling Vibrio parahaemolyticus Biofilms
Source: Viruses. 2022 Jan 27;14(2):264. doi: 10.3390/v14020264 (PMC8879856; doi:10.3390/v14020264)
Supplement: Supplementary file 1 [file viruses-14-00264-s001.zip › Table S1.pdf]

Table S1. Predicted functions of phage FE11 gene products.

Functional modules	Label	Product	Hit	E value	Identidy
DNA metabolism	Gp02	DNA helicase	*Vibrio* phage vB_VpaP_KF1	0	99.77%
	Gp03	DNA helicase	*Vibrio* phage vB_VpaP_KF1	0	99.77%
	Gp04	DNA polymerase	*Vibrio* phage VP93	0	95.44%
	Gp06	nucleotidyl transferase	*Vibrio* phage vB_VpaP_KF1	1.00E-111	78.89%
	Gp09	pyrophosphatase	*Vibrio* phage vB_VpaP_KF1	4.00E-147	97.61%
	Gp12	exonuclease	*Vibrio* phage vB_VpaP_KF1	0	99.68%
	Gp14	endonuclease	*Vibrio* phage vB_VpaP_KF1	5.00E-101	99.32%
	Gp16	deoxynucleoside monophosphate kinase	*Vibrio* phage vB_VpaP_KF1	1.00E-135	95.88%
	Gp18	RNA polymerase	*Vibrio* phage vB_VpaP_KF2	0	99.02%
Structure	Gp21	head-tail connector protein	*Vibrio* phage vB_VpaP_KF2	0	99.80%
	Gp22	scaffolding protein	*Vibrio* phage vB_VpaP_KF1	0	98.89%
	Gp23	capsid protein	*Vibrio* phage vB_VpaP_KF1	0	99.70%
	Gp25	tail tubular protein A	*Vibrio* phage vB_VpaP_KF2	2.00E-133	97.85%
	Gp26	tail tubular protein B	*Vibrio* phage vB_VpaP_KF1	0	95.90%
	Gp27	internal virion protein	*Vibrio* phage vB_VpaP_KF2	1.00E-147	96.04%
	Gp28	internal virion protein	*Vibrio* phage vB_VpaP_KF1	0	98.32%
	Gp30	tail fiber protein	*Vibrio* phage vB_VpaP_KF1	8.00E-144	97.04%
Packaging	Gp32	terminase small subunit	*Vibrio* phage VP93	8.00E-60	98.99%
	Gp33	terminase large subunit	*Vibrio* phage vB_VpaP_KF1	0	99.69%
Lysis	Gp29	peptidoglycan lytic exotransglycosylase	*Vibrio* phage vB_VpaP_KF2	0	99.30%
	Gp31	glycosyl hydrolase	*Vibrio* phage vB_VpaP_KF1	0	97.36%
	Gp38	peptidase M15A	*Vibrio* phage vB_VpaP_KF1	2.00E-107	82.61%
	Gp49	peptidase	*Vibrio* phage vB_VpaP_KF1	0	93.19%
Additional function	Gp07	Fe-S oxidoreductase	*Vibrio* phage vB_VpaP_KF1	0	98.89%
	Gp36	Ig-like domain family protein	*Vibrio* phage vB_VpaP_KF1	4.00E-64	100.00%
