# Supplementary figures and images for: Characterization of the Novel Phage vB_VpaP_FE11 and Its Potential Role in Controlling Vibrio parahaemolyticus Biofilms
Source: Viruses. 2022 Jan 27;14(2):264. doi: 10.3390/v14020264 (PMC8879856; doi:10.3390/v14020264)

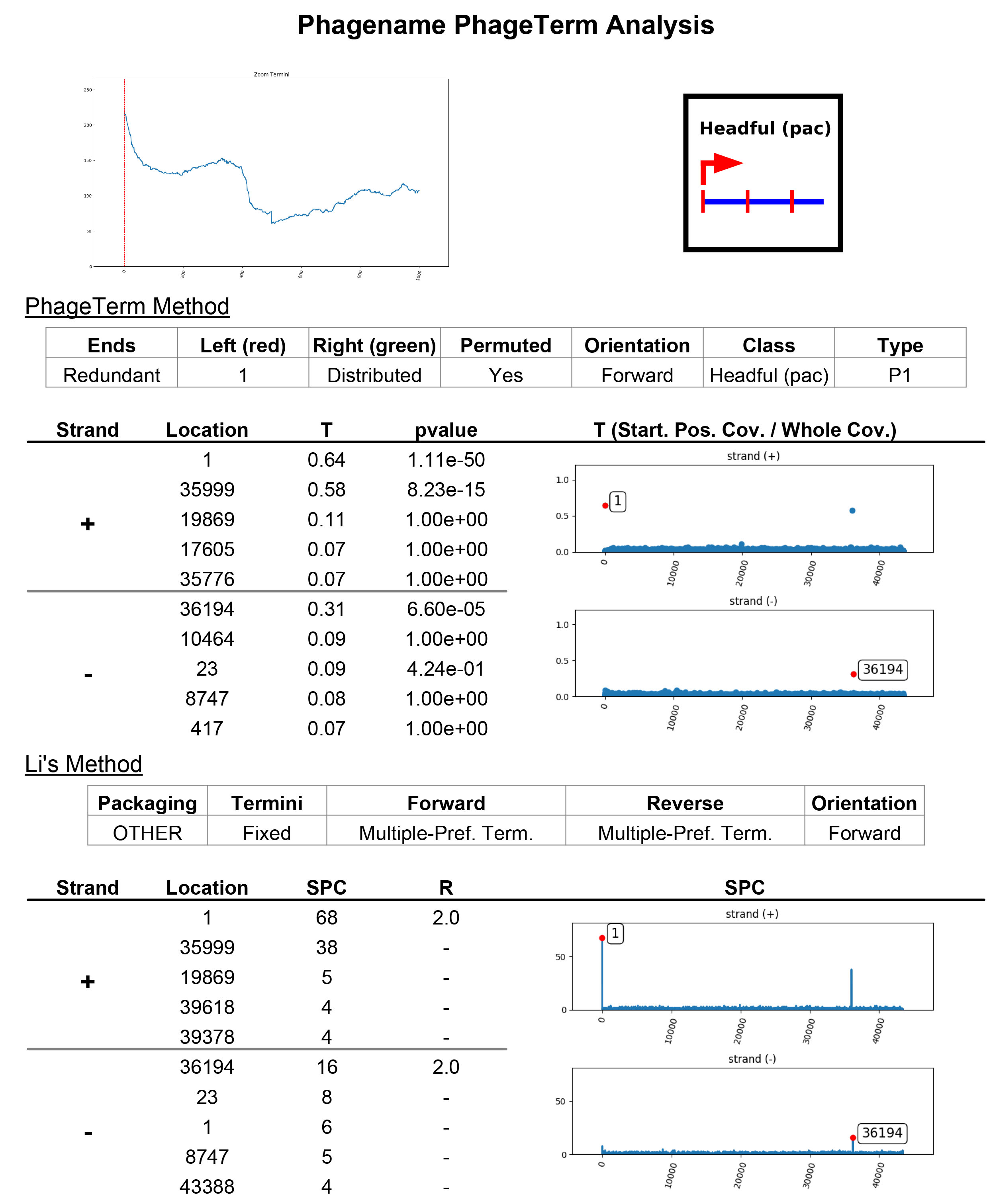

Supplement: Supplementary file 1 [file viruses-14-00264-s001.zip › Figure S1.jpg]
